# Advanced Machine Learning Models for Estimating the Distribution of Sea-Surface Particulate Organic Carbon (POC) Concentrations Using Satellite Remote Sensing Data: The Mediterranean as an Example

**DOI:** 10.3390/s24175669

**Published:** 2024-08-31

**Authors:** Chao Li, Huisheng Wu, Chaojun Yang, Long Cui, Ziyue Ma, Lejie Wang

**Affiliations:** College of Oceanography and Space Informatics, China University of Petroleum (East China), Qingdao 266580, China; 2116020216@s.upc.edu.cn (C.L.); 2016020225@s.upc.edu.cn (C.Y.); z22160008@s.upc.edu.cn (L.C.); 2016020114@s.upc.edu.cn (Z.M.); z22160024@s.upc.edu.cn (L.W.)

**Keywords:** particulate organic carbon, Mediterranean, machine learning, tuneRanger R package, geographic detector

## Abstract

Accurate estimation of the distribution of POC in the sea surface is an important issue in understanding the carbon cycle at the basin scale in the ocean. This study explores the best machine learning approach to determine the distribution of POC in the ocean surface layer based on data obtained using satellite remote sensing. In order to estimate and verify the accuracy of this method, it is necessary to obtain a large amount of POC data from field observations, so this study was conducted in the Mediterranean Sea, where such data have been obtained and published. The research initially utilizes the Geographic Detector (GD) method to identify spatial correlations between POC and 47 environmental factors in the region. Four machine learning models of a Bayesian optimized random forest (BRF), a backpropagation neural network, adaptive boosting, and extreme gradient boosting were utilized to construct POC assessment models. Model validation yielded that the BRF exhibited superior performance in estimating sea-surface POC. To build a more accurate tuneRanger random forest (TRRF) model, we introduced the tuneRanger R package for further optimization, resulting in an R^2^ of 0.868, a mean squared error of 1.119 (mg/m^3^)^2^, and a mean absolute error of 1.041 mg/m^3^. It was employed to estimate the surface POC concentrations in the Mediterranean for May and June 2017. Spatial analysis revealed higher concentrations in the west and north and lower concentrations in the east and south, with higher levels near the coast and lower levels far from the coast. Additionally, we deliberated on the impact of human activities on the surface POC in the Mediterranean. This research contributes a high-precision method for satellite retrieval of surface POC concentrations in the Mediterranean, thereby enriching the understanding of POC dynamics in this area.

## 1. Introduction

Various models have been proposed by researchers to estimate POC using satellite remote sensing, and each is based on the relationship between POC and intrinsic optical properties (IOPs), apparent optical properties (AOPs), and other characteristics related to POC. For instance, Fellous and colleagues used total particulate absorption (ap) as a POC proxy. Based on measurements in the Southern Ocean, Stramski et al. [1] found a close correlation between the POC concentration and the optical backscattering of particles suspended in seawater. Building upon this principle, they developed an algorithm to estimate surface POC using ocean color satellite data [1]. Woźniak et al. [2] derived a new equation for estimating the concentration of suspended particulate matter (SPM) and POC in surface water in the southern Baltic Sea using spectral values of remote sensing reflectance. In estimating POC in the Gulf of Mexico, Son et al. [3] achieved exceptional results by introducing the Maximum Normalised Difference Carbon Index (MNDCI), which is based on the maximum band ratio of blue-green wavelengths. In addition to the MNDCI algorithm, other methodologies utilize the relationship between POC and AOPs to estimate POC. Based on the empirical relationship between POC and the blue-green band ratio of reflectance, RRS(λB)/RRS (555), Stramski et al. developed a two-step algorithm. This algorithm uses relationships linking reflectance and POC with IOPs to invert POC [4]. Utilizing the Color Index (CI), an algorithm for estimating POC was developed by Le et al. [5]. Different algorithms are required for different marine environments, such as open ocean (type I) and coastal waters (type II). Integration of these algorithms to achieve optimal performance has attracted much attention from researchers. A combined algorithm was developed by Stramski et al. based on the Band Ratio Difference Index (BRDI) and the MBR-OC4 algorithm for POC retrieval [6]. It maintains high accuracy in both Type I and Type II waters. Algorithms for POC retrieval in the East China Sea were investigated by Le et al. (2022) [5] utilizing the CI and band ratio algorithms. Their results, obtained through temporal series analysis, demonstrated satisfactory accuracy [7,8].

In addition to traditional methods, many researchers have also attempted to build models using machine learning techniques. Raphaelle et al. utilized artificial neural network models to estimate the vertical distribution of the backscattering coefficient of particles (BBP) globally. Significant improvements in both accuracy and product resolution were observed compared to previous methodologies [9]. Several machine learning algorithms, including k-Nearest Neighbors (KNNs), gradient boosting, random forest (RF), AdaBoost, and Partial Least Squares Regression (PLS), were used by Fellous et al. to estimate the sea surface concentration of POC in the Mediterranean. The resulting R^2^ values were 73.84%, 72.33%, 74.70%, 61.5%, and 50.12% [10]. The configuration of hyperparameters directly impacts the performance of machine learning models. Thus, selecting an appropriate tuning method is crucial. Bayesian optimization (BO) is an efficient tuning method [11,12]. Based on the principle of Sequential Model-Based Optimization (SMBO), the tuneRanger R package (TR) was successfully investigated by Probst et al. using out-of-bag prediction, which was specifically designed to tune the parameters of RFs [13].

These machine learning models are promising for estimating the distribution of POC concentration in the sea surface layer using satellite remote sensing. However, it is also important to determine what available data are suitable for input to the machine learning model. Geographic detector (GD) is a spatial analysis method utilized to detect spatial heterogeneity and reveal the underlying driving forces. It is widely employed when conducting driving force analysis and factor analysis. GD quantifies spatial heterogeneity by statistically evaluating variance [14]. Therefore, in this study, we innovatively adopt the GD method to select factors for model training. In addition, a large number of POC data from field observations are also required for training and accuracy assessment of machine learning models. Therefore, the study was conducted in the Mediterranean Sea, where such data have been obtained and published. In this study, we use several machine learning models: a Bayesian optimized random forest (BRF), a backpropagation neural network (BPNN), adaptive boosting (AdaBoost) and extreme gradient boosting (XGBoost). To build a more accurate the tuneRanger random forest (TRRF), we innovatively introduced the tuneRanger R package.

## 2. Materials and Methods

### 2.1. Study Area

The Mediterranean Sea, a semi-enclosed region characterized by high salinity, elevated temperatures, and dense waters, experiences net evaporation surpassing precipitation. This leads to the phenomenon of anti-estuarine circulation at the Strait of Gibraltar, resulting in notably low nutrient concentrations. Due to the high population density surrounding the Mediterranean, it exhibits high sensitivity to anthropogenic influences [15]. The concentration of POC in surface waters is intricately linked to organic carbon excretion, remineralization, biological production, and oceanic internal export and significantly impacts the nutrient concentrations in the Mediterranean [16]. Following the sinking of POC from surface waters, it serves as a biological pump, facilitating the storage of carbon in the deep sea [4]. This study attempted to estimate the distribution of POC concentrations at the basin scale using a novel approach that used machine learning. In the Mediterranean, large amounts of satellite imagery data and in situ POC observation data are available. Therefore, this approach was applied to determine the distribution of POC concentrations at the basin scale in the Mediterranean.

### 2.2. Data Sources

#### 2.2.1. In Situ Data

**Figure 1:**
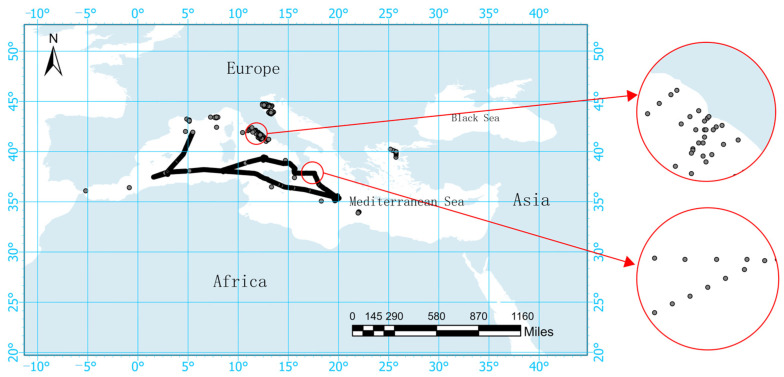
Observed particulate organic carbon data in the Mediterranean from 15 May 2017 to 10 June 2017 (shown in gray). To gain a detailed understanding of the observation points, locations both near the coast and far from the coast were selected for a localized examination.

The measured data were from the SeaWiFS Bio-optical Archive and Storage System website (https://seabass.gsfc.nasa.gov/ (accessed on 23 June 2024)), which includes measured POC data from the Mediterranean. The data collection period spanned from 15 May 2017, to 10 June 2017. SeaBASS is a local repository utilized by NASA’s Ocean Biology Processing Group (OBPG) for satellite validation purposes [17]. The SeaWiFS and SIMBIOS project offices have established requirements for in situ data and sampling strategies to ensure the acceptability of observational results for algorithm development purposes. In situ data collected at depths of less than 10 m were selected to ensure the accuracy of the results. After screening, a total of 11,706 measurements of surface POC in the Mediterranean were obtained. The distribution of the measurement points is depicted in Figure 1, illustrating that the concentration of POC was higher near the coast compared to far from the coast.

#### 2.2.2. Satellite Data

Remote sensing data and reanalysis data obtained from multiple databases were downloaded from the Copernicus Marine Service (https://marine.copernicus.eu/ (accessed on 23 June 2024)). Considering the collection times of the observed POC data and the absence of corresponding satellite data in certain regions at certain times, data from 10 May 2017 to 15 June 2017 were selected. The products mainly included 11 bands (412 nm, 443 nm, 490 nm, 555 nm, 670 nm, 547 nm, 645 nm, 667 nm, 469 nm, 488 nm, and 510 nm) that indicated remote sensing reflectance (Rrs), chlorophyll a (Chl), oxygen (O_2_), silicate (SiO_3_), nitrate (NO_3_), phosphate (PO_4_), seawater salinity (SSS), euphotic zone depth (ZEU), pH, ocean mixed-layer thickness (Mld), geostrophic eastward ocean velocity (Ugos), geostrophic northward ocean velocity (Vgos), sea-surface temperature (SSTemp), sea-surface density (Dos), the volume absorption coefficient of radiative flux in seawater due to dissolved organic matter and non-algal particles (CDM), the backscattering coefficient of particles (BBP), the diffuse attenuation coefficient at 490 nm (Kd_490), and suspended particulate matter (SPM). Details of these parameters, including their full names, source datasets, spatial resolutions, and time resolutions and the amounts of collected remote sensing and reanalysis data, are summarized in Table 1.

#### 2.2.3. Agreement between In Situ and Satellite Data

For this study, we developed a program that performed spatiotemporal matching between remote sensing data and measured POC data. This program averaged the POC concentrations that appeared in the same spatial modeling units. A total of 1532 matched data points were obtained for the period from 15 May 2017 to 10 June 2017. The data were divided into training, validation, and testing sets at a ratio of 6:2:2, respectively. These sets were used for training, parameter tuning, and testing the model’s performance on the data used in this study. This approach enhanced the accuracy of the training and evaluation of the machine learning algorithms. The maximum observed POC concentration was 57.35 mg/m^3^. The minimum was 20.68 mg/m^3^, and the average was 30.59 mg/m^3^.

### 2.3. Feature Selection

In this study, GD was employed as the method for feature selection. The primary concept behind GD involves dividing a study area into different subregions based on various variables. It then compares the variances between the different subregions and within each subregion to assess the explanatory power of potential variables [18]. The GD model eliminates a linear assumption. Thus, the results obtained are not affected by collinearity among multiple variables [19]. Spatial data discretization is crucial for identifying feature variables. Before implementing the GD model, data discretization is performed as a preliminary step [20]. The q-value is used to represent the extent to which a variable can explain the spatial variation of the dependent variable. The formula is as follows:

(1) $$q=1-\frac{\mathrm{SSW}}{\mathrm{SST}}$$

In this equation, SSW stands for within sum of squares and SST stands for total sum of squares. The q-value ranges from 0 to 1, where a higher value indicates a stronger correlation between the influencing factor and POC.

### 2.4. Model Selection

XGBoost is an extensible end-to-end tree-boosting system. To estimate the suspended particulate matter in global lakes, an XGBoost model was trained by Wen et al. [21]. To invert global POC concentrations, an XGBoost model was also trained by Liu et al. [22]. In this study, the following optimal hyperparameters were selected: the number of trees was 192, the learning rate was set to 0.1, the maximum depth per tree was 10, and the minimum weight per leaf node was 9.798.

RF was first introduced by Breiman in 2001 [23]. Today, RF is widely applied in remote sensing analysis [24] to reduce overfitting and overlearning and maintain the accuracy of results [23]. Recent studies have shown that RF achieves higher accuracy compared to other multivariate linear regression models when considering multiple variables [25,26]. In this study, the following parameters were selected: the minimum samples per leaf node was 1, the maximum depth was 17, the minimum samples split was 20, and the number of trees was 275.

AdaBoost is an excellent boosting algorithm that can improve the accuracy of weak learning algorithms [27]. The parameters of the AdaBoost model used in this study were as follows: the learning rate was 1 and the number of trees was 181.

An ANN is a complex network structure consisting of input layers, hidden layers, and output layers [28]. A BPNN utilizes gradient descent to continuously adjust the network’s weights and thresholds through backpropagation, aiming to minimize the sum of squared errors in the network. The parameters of the BPNN model used in this study were as follows: 1 input layer, 10 hidden layers, and 1 output layer. The first hidden layer consisted of 89 neurons, while the remaining layers consisted of 52 neurons each.

### 2.5. Dual Optimization of the RF Model

#### 2.5.1. Initial Optimization of the RF Model

BO [29,30,31] is a global optimization method based on sequential models. First, a probability model is constructed by defining distributions based on the objective function. Then, this model is refined [32]. Unlike the other two commonly used tuning methods, grid search and random search, BO utilizes previously searched points to determine the next search point and is used to solve low-dimensional black-box optimization problems. The core of this algorithm is modeling the objective function using Gaussian process regression and constructing an acquisition function to determine the locations of the sampled points. Gaussian process predictors are widely used in Bayesian methods, requiring O(n² space and O(n³ time for the use of n datasets [33]. Since the performance of an RF is influenced by numerous hyperparameters, including the number of trees, the maximum depth of each tree, and the minimum sample number for each node, the BO algorithm was incorporated into an RF to enhance model accuracy, resulting in the BRF used in this study.

#### 2.5.2. Re-Optimization of the RF Model

Building on the BO method, TR was used to fine-tune the parameters of the BRF model, resulting in the TRRF model used in this research, which was then compared to the BRF model. TR is based on the R packages ranger and mlrMBO. Its principle involves employing SMBO as an adjustment strategy [34]. This function simultaneously adjusts the randomly selected candidate variables (mtry), sample size, and node size using out-of-bag prediction as the evaluation method, which is much more efficient than using cross-validation. The final selection of hyperparameters involves selecting the top 5% from all SMBO iterations, calculating the average value for each hyperparameter, and rounding for mtry and node size [12]. After iterations, mtry was set to 2, the min node size was set to 2, and the sample fraction was set to 0.898.

### 2.6. Statistical Indicators Used for Model Development, Validation, and Testing

Bias, variance, goodness of fit (R^2^), root-mean-square error (RMSE), mean absolute percentage error (MAPE), mean absolute error (MAE), and mean squared error (MSE) were used as metrics to measure the performance of the model.

Bias is the error between a model’s predicted values and the true values. It describes the overall error direction of a model and characterizes the fitting ability of the learning algorithm itself. Its formula is as follows:

(2) $$\mathrm{Bias}=\frac{1}{n}\times \sum \left({\mathrm{POC}}_{\mathrm{pre}}-{\mathrm{POC}}_{\mathrm{true}}\right)$$

Variance describes the dispersion of the predicted values, that is, the distances between them and the expected values. The larger the variance, the more scattered the data distribution. Its formula is as follows:

(3) $$\mathrm{Variance}=\frac{1}{n}\times \sum {\left({\mathrm{POC}}_{\mathrm{pre}}-{\mathrm{POC}}_{\mathrm{mean}}\right)}^{2}$$

R^2^ describes the degree to which a model fits the data, with 0 ≤ R^2^ ≤ 1. If the result is 0, it indicates that the model fits the data poorly; if the result is 1, it means the model is error-free. In general, the larger the R^2^, the better the fit of the model. The formula for calculating R^2^ is as follows:

(4) $$R^{2}=1-\frac{\mathrm{SSR}}{\mathrm{SST}}$$

In this equation, SSR represents the sum of squares due to regression and SST stands for total sum of squares.

The RMSE describes the deviation between predicted values and actual values and is sensitive to outliers in the data. Its calculation formula is as follows:

(5) $$\mathrm{RMSE}=\sqrt{(\frac{1}{n}\times \sum {\left({\mathrm{POC}}_{\mathrm{pre}}-{\mathrm{POC}}_{\mathrm{true}}\right)}^{2})}$$

The MAPE is sensitive to relative errors and does not change with proportional changes in the target variable, making it suitable for data with large differences in the scales of the target variables. Its calculation formula is as follows:

(6) $$\mathrm{MAPE}=\frac{1}{n}\times \sum \left(\left|\frac{{\mathrm{POC}}_{\mathrm{pre}}-{\mathrm{POC}}_{\mathrm{true}}}{{\mathrm{POC}}_{\mathrm{true}}}\right|\right)\times 100$$

The MAE represents the average of the absolute errors between observed and predicted values. It is a linear score that treats all individual differences equally without disproportionately magnifying high differences. Consequently, it is not sensitive to outliers. Its calculation formula is as follows:

(7) $$\mathrm{MAE}=\frac{1}{n}\times \sum \left|{\mathrm{POC}}_{\mathrm{pre}}-{\mathrm{POC}}_{\mathrm{true}}\right|$$

The MSE represents the average of the squared differences between observed and predicted values. It is useful for measuring the average error and can evaluate the degree of variation in data, thereby indicating the accuracy of a predictive model. Its calculation formula is as follows:

(8) $$\mathrm{MSE}=\frac{1}{n}\times \sum {\left({\mathrm{POC}}_{\mathrm{true}}-{\mathrm{POC}}_{\mathrm{pre}}\right)}^{2}$$

To measure the model’s fit, two indicators were selected for this study: R^2^ and bias. To assess the predictive variability of the model across different samples, the RMSE, variance, MSE, and MAE were chosen. To describe the overall error of the model, the MAPE was selected.

## 3. Results and Discussion

### 3.1. Feature Selection

In this study, the GD method was employed for factor detection and variable factors were selected for model training. These factors could be categorized into three types.

The first category comprised AOPs and their mathematical combinations. AOPs are quantities sensitive to lighting conditions, including downward irradiance (Ed), upward irradiance (Eu), water-leaving radiance (LW), Rrs, radiance ratios, and their respective diffuse attenuation coefficients. These parameters are crucial for ocean remote sensing, as they are affected by the absorption and scattering of light in seawater [35]. In this category, the volume attenuation coefficient of downwelling radiative flux in seawater (Kd_490) and the Rrs at wavelengths ranging from 412 nm to 670 nm, encompassing the red, green, and blue wavelength regions, were collected. Based on the combination of Rrs values at these wavelengths, various spectral indices and ratios were computed, including band ratios (e.g., red/green, red/blue, and blue/green), CI, the normalized difference carbon index (NDCI), the band ratio difference index (BRDI), the MNDCI, and the maximum band ratio (MBR) [6].

The second category encompassed features potentially associated with POC. This study included Chl, O_2_, SiO_3_, NO_3_, PO_4_, SSS, euphotic zone depth (EP), pH, Mld, Ugos, Vgos, SSTemp, and DOS [36]. The chlorophyll-a concentration serves as an indicator of photosynthesis intensity and phytoplankton biomass in the ocean. The oxygen concentration influences microbial respiration, thereby impacting the remineralization process of POC and subsequently affecting its concentration on the ocean surface. In oxygen-depleted zones, a considerable portion of the POC generated through surface ocean photosynthesis may sink into the deep sea, further influencing the surface POC concentration [37]. Silicates significantly influence the production of particulate carbon by phytoplankton [38]. The concentration of POC greatly influences the content of bioavailable phosphorus on the ocean surface because POC serves as a carrier of endogenous phosphorus [39]. Sea-surface salinity and temperature exert direct and indirect effects on the growth and reproduction of marine surface flora and fauna. EP is closely linked to the photosynthesis of surface marine plants. pH significantly influences various chemical processes by creating different chemical environments. Changes in MLD result in variations in the distribution of various nutrient salts. The dissolved oxygen distribution, including variations due to light penetration, also impacts the concentration of POC on the surface of the ocean.

The third category comprised IOPs, which are solely influenced by water composition and remain constant regardless of changes in light conditions. IOPs encompass various components such as the absorption and attenuation coefficients within water. In this study, the backscattering coefficient of particles was selected from the IOPs as a candidate feature.

**Figure 2:**
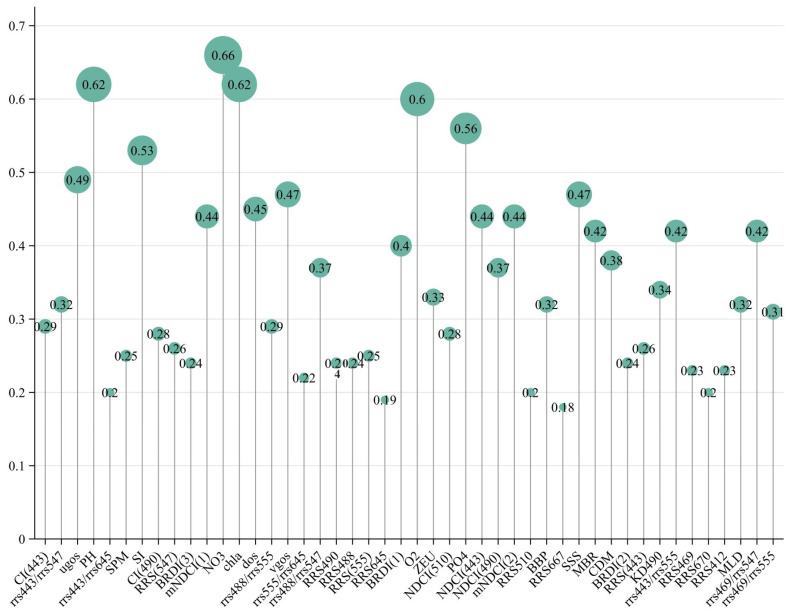
The q-values of the different features were obtained using the geographic detector.

Following the determination of the range of study features, this research employed the R language package developed by Song et al. for geographical detection [18]. Before employing this method, it was necessary to discretize the continuous variables. The GD package offers four discretization methods: equal intervals, geometric intervals, quantiles, and natural breaks. The factor detection results are depicted in Figure 2. The interaction detection results suggested either bivariate or nonlinear enhancement, both of which were selected. In the factor detection results, variables with q < 0.45 were regarded as weakly correlated with surface POC, whereas those with q > 0.45 were deemed to be strongly correlated. Finally, ten variables were selected to train the model used to estimate sea-surface POC, including the MNDCI(I), NDCI (443), and Kd_490 from the AOPs and their mathematical combinations; BBP from the IOPs; and other features related to POC such as NO_3_, O_2_, PO_4_, SPM, SSS, and Chl. Selection was based on excluding factors with higher q-values that were not mechanistically related to POC. The oxygen concentration affects microbial respiration, which in turn influences the remineralization of POC, thus affecting the surface POC concentration in the ocean. POC can increase the rate of nitrogen mineralization and can also affect the denitrification and nitrate retention rates in the ocean [37]. It also serves as a carrier of endogenous phosphorus, and its concentration significantly impacts the availability of phosphorus to surface marine organisms [39]. The chlorophyll concentration reflects the intensity of photosynthesis and the biomass of oceanic plants. Sea-surface salinity directly or indirectly influences the growth and reproduction of marine organisms. Suspended particulate organic matter serves as a crucial carbon source in the ocean, contributing to increases in POC. All these factors play significant roles in the formation and transformation of POC. In Huizeng Liu et al.s’ global ocean study [22], the selected factors included Rrs490/Rrs555, Rrs490/Rrs510, Rrs510/Rrs555, Kd (490), the CI, and chlorophyll a. Similar to the MNDCI(I) and NDCI (443) selected in this study, these were combinations of RRS from different spectral bands. Additionally, both studies utilized Kd_490 and chlorophyll a, which partially validated the innovative use of geographic detectors in this study. Huisheng Wu et al.s’ research [40] included factors such as Rrs (469), Rrs (547), Rrs (555), Rrs (667), Kd490, Rrs (443)/Rrs (555), Rrs (443)/Rrs (557), Rrs (488)/Rrs (555), Rrs (488)/Rrs (547), Rrs (469)/Rrs (555), Rrs (469)/Rrs (547), the CI (488), the NDCI (488), and the BRDI. These were also combinations of RRS from different bands. Additionally, they used factors like BBP, SSTemp, SSS, CHL, EZD, and MLD, which were similar to the factors of BBP, SPM, SSS, NO_3_, and PO_4_ used in this study. GD is an effective method for identifying spatial correlations between factors and variables, but it has rarely been used in POC-related research. This study innovatively applied this method to select factors influencing the POC concentration in the Mediterranean. Comparative analysis with other studies using different methods to select influencing factors indicated that this approach yielded favorable results.

### 3.2. Machine Learning Models for POC

#### 3.2.1. Accuracy of the Models for Different Datasets

The BO method was employed to adjust the hyperparameters of the RF and XGBoost models, whereas the parameters of the AdaBoost and BPNN models were set manually.

**Figure 3:**
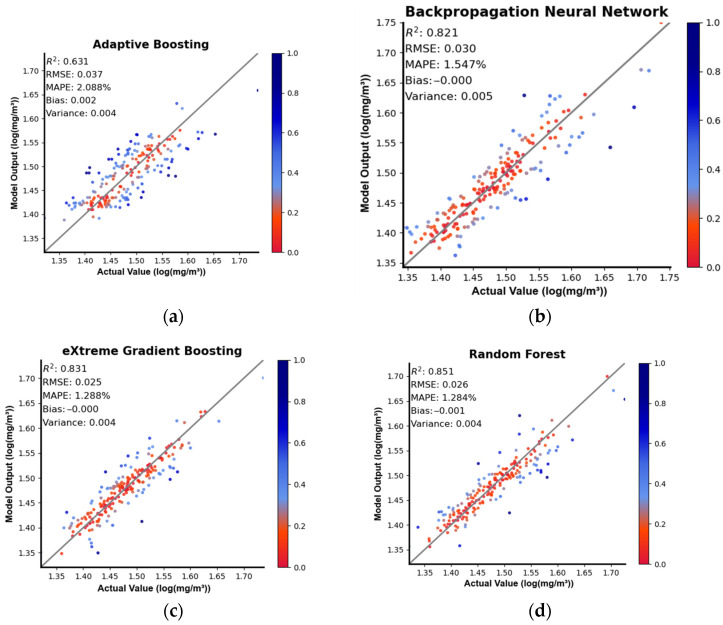
Scatterplots describing the results of the models, with the colors of the dots representing the magnitudes of the normalized residuals (Subfigures (**a**–**d**) show the performance of the four models: Adaptive Boosting, Backpropagation Neural Network, eXtreme Gradient Boosting, and Random Forest, on the dataset. The vertical axis represents the model-predicted POC concentration values, while the horizontal axis represents the actual POC concentration values).

The BRF, XGBoost, AdaBoost, and BPNN models demonstrated strong fitting capabilities for nonlinear functions and excelled in completing multivariate regression tasks. To enhance the models’ generalization ability, we transformed the target variable, the surface POC in the ocean, by taking the base-10 logarithm as the model input. Table 2 presents the evaluation metrics of the four machine learning models for the training, validation, and test sets, where the metrics highlighted in bold represent the best performance of each model for the corresponding dataset. Among the four models, the BRF model exhibited the best performance, with a bias of −0.001, a variance of 0.004, an R^2^ of 0.85, an RMSE of 0.026 log(mg/m^3^), and a MAPE of 1.284% for the test set. Therefore, when estimating the concentration of surface POC in the Mediterranean Sea, the BRF model exhibited strong superiority in both generalization ability and fitting degree. The partitioning of water bodies based on the contribution of organic particles to suspended particles showed that the surface water in the Mediterranean Sea is predominantly organic in nature. Previous studies have similarly highlighted the significant advantages of the RF algorithm over other machine learning algorithms in estimating the concentration of POC in organic water [40]. The BRF algorithm is an optimized version of the RF algorithm, and this explains why it outperforms other algorithms in estimating the concentration of surface POC in the Mediterranean Sea. We utilized normalized residuals to detect model outliers and assess the fitting degrees of the models. In this study, we created scatter plots of the normalized residuals of the predictions from the four models for the test set, as shown in Figure 3, where the colors of the points reflect the magnitudes of the normalized residuals. In the scatter plot, it is apparent that the AdaBoost algorithm had poor fitting compared to the other three algorithms, while the RF algorithm exhibited the best fitting with an R^2^ of 0.851. The data used in this study were relatively concentrated, mostly ranging from 20 to 40 mg/m^3^. This suggests that for predicting low concentrations of POC, the BRF, XGBoost, and BPNN models are all suitable, with the BRF model performing the best.

#### 3.2.2. Re-Optimization of the RF Model

To further optimize the model for higher accuracy, we employed TR, developed by Probst et al., which was specifically designed to tune the parameters of RFs. SMBO was utilized as the tuning strategy, and out-of-bag prediction was used for evaluation [13]. The evaluation metrics included R^2^, MAE, and MSE. The precision results were compared when adjusting the hyperparameters of the RF using TR and Python’s BO method. The results are presented in Table 3.

In Table 3, it is observed that the TRRF model outperformed the BRF model in terms of the R^2^, MSE, and MAE metrics. Besides achieving higher accuracy, the TRRF utilized out-of-bag prediction as the evaluation method during parameter tuning, which was notably faster compared to using cross-validation or splitting the dataset for evaluation [12], achieving improvements in both accuracy and speed. Compared to the other models used in this study, the TRRF model performed significantly better, and it was the best method developed in this study for predicting POC in the Mediterranean. In a study by Fellous et al. on estimating the POC concentration in the Mediterranean [10], models such as k-Nearest Neighbors (KNNs) and gradient boosting (GB) were employed, and an accuracy (R^2^) of 0.73 was achieved. However, in this study, the TRRF model that was ultimately selected achieved a higher accuracy, with an R^2^ of 0.87. Apart from the innovative use of geographic detectors when selecting influential factors, another significant reason for this improvement was the TRRF model’s effectiveness in estimating the POC concentration in the Mediterranean. Therefore, in this study we ultimately decided to use the TRRF model for inversion.

Table 4 presents the main findings of this study and compares them with those of the previous study.

### 3.3. Assessment of the TRRF Model

#### 3.3.1. Comparison of TRRF Products and NASA Products

A comparison was made between the Mediterranean POC estimation products obtained from the TRRF and the POC products retrieved using NASA’s band ratio algorithm for May and June 2017. POC products for May and June 2017 were downloaded from NASA OCEAN COLOR, and the deviations as well as percentage deviations between the products derived from the two algorithms were calculated.

**Figure 4:**
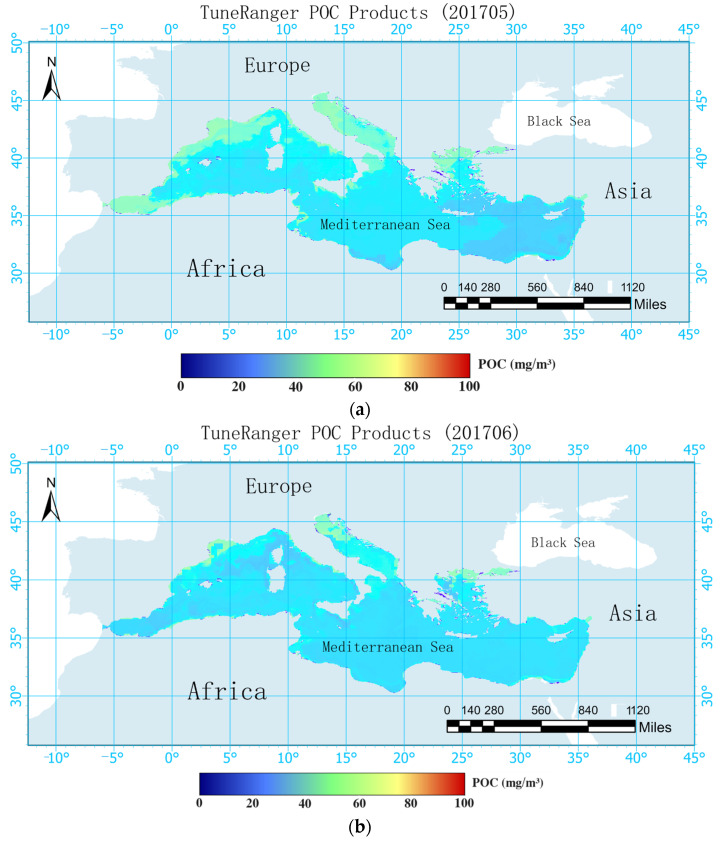

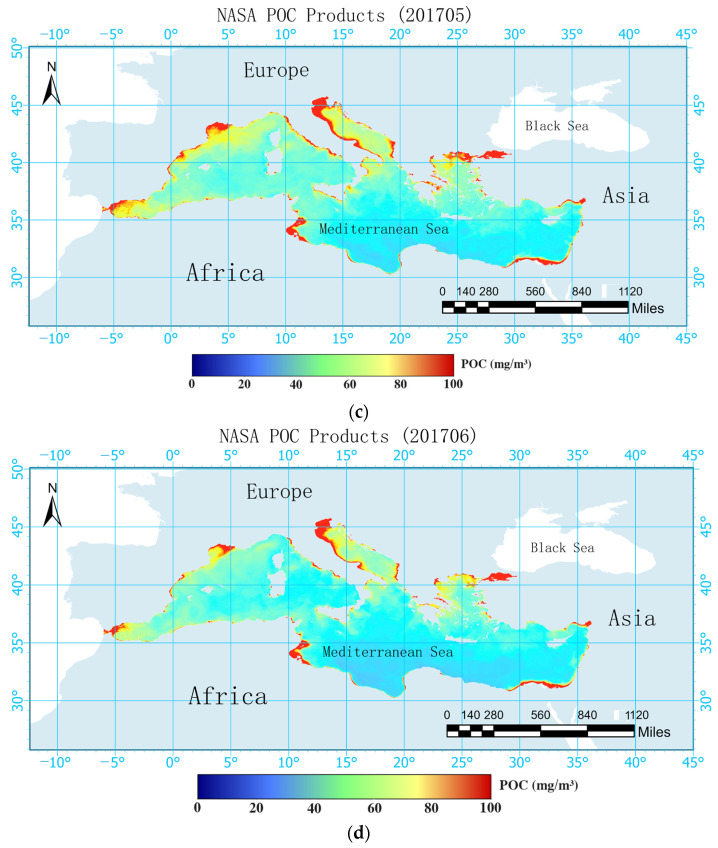
Comparison of products of the random forest algorithm optimized with the tuneRanger R package and NASA’s POC products in the Mediterranean in May and June 2017 (Subfigures (**a**,**b**) in the figure show the POC concentrations predicted by the TRRF model for the Mediterranean region in May and June 2017, respectively. Subfigures (**c**,**d**) show the POC concentrations predicted by NASA for the same region in May and June 2017).

The comparison illustrated in Figure 4 reveals that the algorithm employed by NASA and the TRRF model used in this study exhibited similar spatial distributions of POC. POC demonstrated a pattern of higher concentrations in the north and west, lower concentrations in the south and east, and elevated concentrations near the coast compared to far from the coast. Given the geographical positioning of the Mediterranean, it is apparent that Europe, situated to its west and north, experiences greater economic development in comparison to Africa to its south and Asia to its east. Rapid economic development can contribute to heightened environmental pollution, leading to elevated levels of nitrogen, phosphorus, and other nutrients in the water, which may result in phenomena such as algal blooms. Increases in nutrients such as nitrogen and phosphorus have led to the proliferation of marine microorganisms and improved the production of POC. This phenomenon explains the distribution pattern where POC concentrations are higher in the northern and western parts of the Mediterranean compared to the southern and eastern parts. Many nutrients accumulate near the coast, with some diffusing further, resulting in higher POC concentrations near the coast than far from the coast. Over 80% of the POC concentrations fell within the range of 0–50 mg/m^3^, with only a portion estimated by NASA exceeding 50 mg/m^3^ in coastal areas.

**Figure 5:**
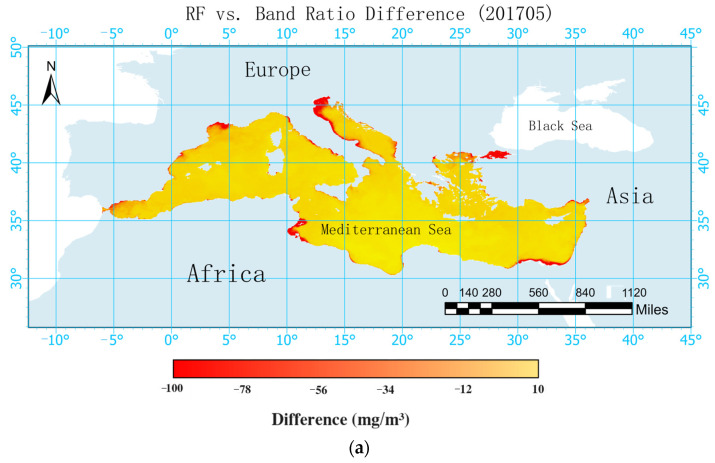

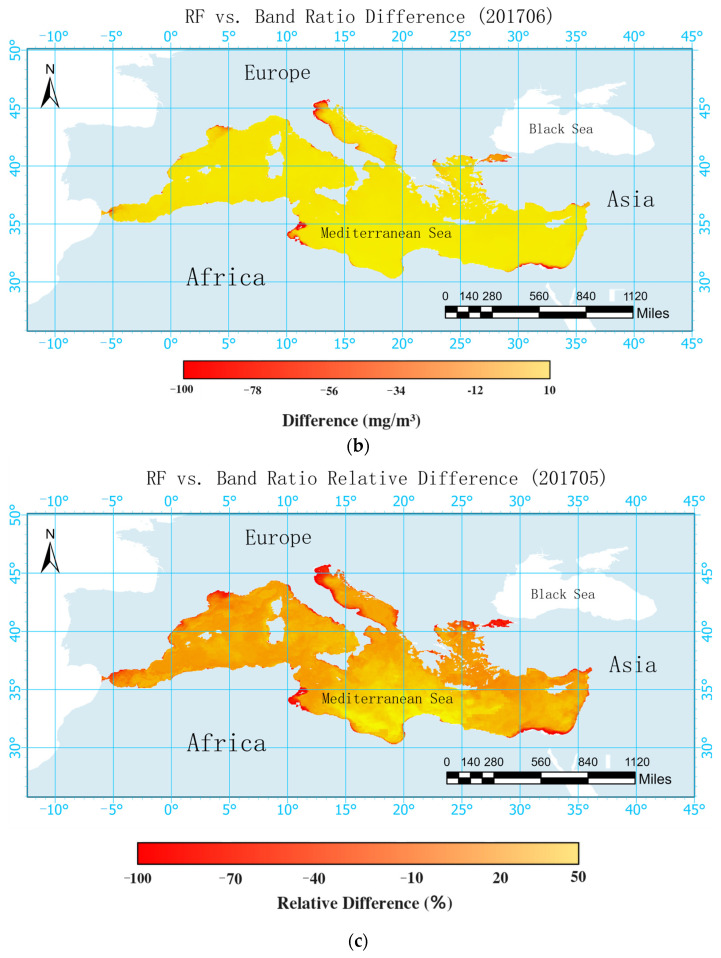

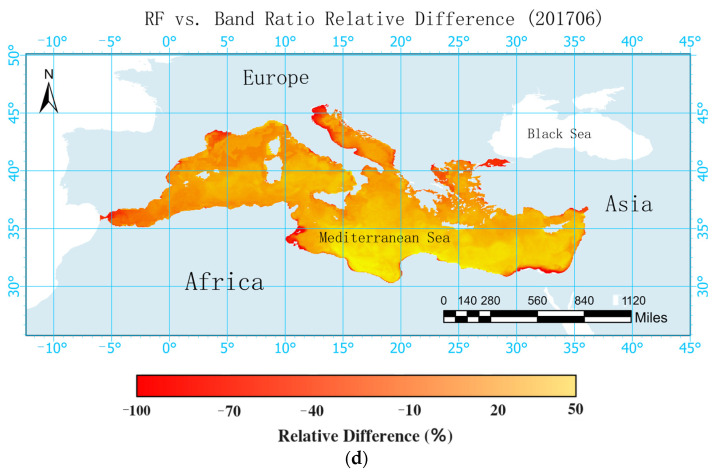
Deviations and percentage deviations of products retrieved by random forest algorithm optimized with tuneRanger R package and band ratio algorithm in May and June 2017 (Subfigures (**a**,**b**) in the figure show the differences between the final products of this study and the products obtained using the band ratio algorithm for May and June 2017, respectively. Subfigures (**c**,**d**) show the percentage differences between the final products of this study and the products obtained using the band ratio algorithm for May and June 2017, respectively).

Overall, the POC concentrations estimated using the TRRF model were lower than those estimated by NASA for the Mediterranean. Figure 5 depicts the deviations and percentage deviations of the Mediterranean POC products estimated by the TRRF and the band ratio algorithm employed by NASA for May and June 2017. In May 2017, deviations between the two products of less than 10 mg/m^3^ accounted for 82.29% of the data and deviations of less than 20 mg/m^3^ accounted for 96.16% of the data, with only a few coastal areas exhibiting deviations greater than 20 mg/m^3^. Comparing the percentage deviations of the two products, it was found that 81.80% of the areas had percentage deviations of less than 20%, 92.25% had percentage deviations of less than 30%, and 98.03% had percentage deviations of less than 50%. In June 2017, deviations between the two products of less than 10 mg/m^3^ accounted for 87.11% of the data, deviations of less than 20 mg/m^3^ accounted for 96.47% of the data, and a small number of coastal areas had deviations greater than 20 mg/m^3^. For the percentage deviations, 76.66% of the areas deviated by less than 20%, 93.16% deviated by less than 30%, and 98.05% deviated by less than 50%. In terms of time dynamics, the deviation in May 2017 was slightly larger than that in June 2017, while the percentage deviation was almost the same. Generally, there are no significant changes in the marine environment of the Mediterranean Sea within two consecutive months, so it is reasonable that the deviations and percentage deviations obtained by the two algorithms were almost consistent over these two months. From a spatial perspective, the distribution of POC demonstrated the characteristics of higher concentrations in the north and west, lower concentrations in the south and east, and higher concentrations near the coast compared to far from the coast. In addition, the deviations and percentage deviations of the products exhibited similar patterns. This was attributed to the fact that both the TRRF and the band ratio algorithm are less accurate when estimating POC in complex marine environments compared to simpler environments. When estimating POC in environments with high nutrient contents and intense human activities, significant errors may occur, which do not follow a specific pattern. Therefore, the deviations and percentage deviations of the two products may become relatively large due to the accumulation of errors.

#### 3.3.2. Comparison between TRRF Products, NASA Products, and Actual Measured Values

**Figure 6:**
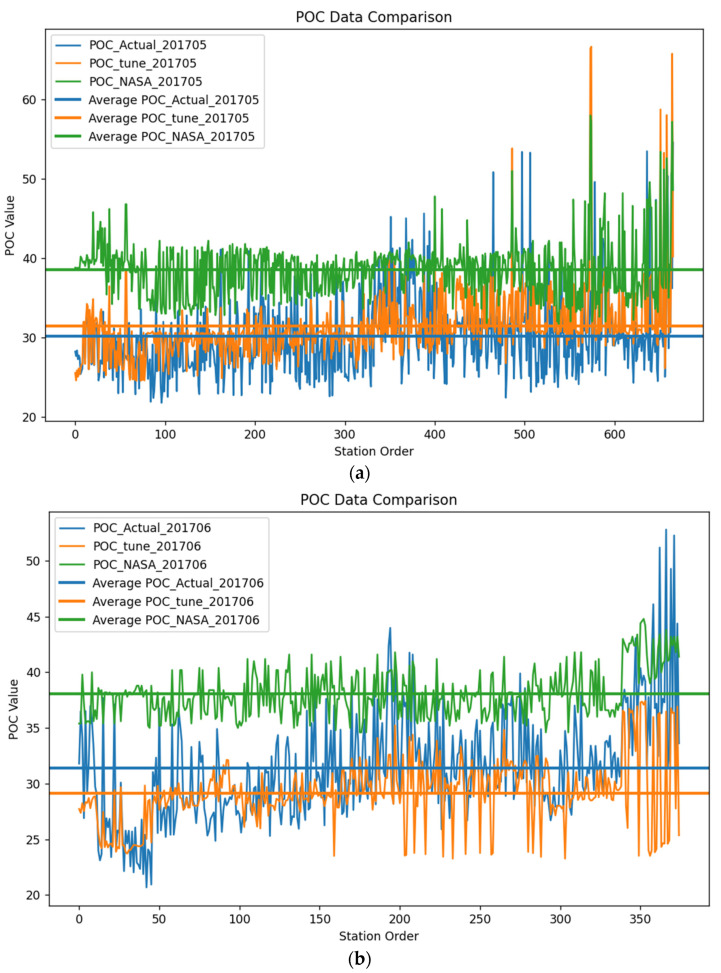
Comparison of actual particulate organic carbon (POC) measurements, POC concentrations obtained by the random forest algorithm optimized with the tuneRanger R package, POC concentrations from NASA, and their average values at the in situ observation locations in May and June 2017 (Subfigures (**a**,**b**) show comparisons between the actual POC values, TRRF model predictions, and NASA predictions for May and June 2017, respectively. The line plots represent the data values for each site, while the straight lines indicate the average values for each result).

In Figure 6, the lines represent the POC values at different measurement stations, while the horizontal lines represent the average values of the measured values and the two products. It can be observed that the POC values obtained by the TRRF were closer to the measured values in May and June 2017, while the NASA product tended to overestimate the surface POC concentration in the Mediterranean Sea to a greater extent. This indicates that the products obtained in this study are more suitable for estimating the surface POC in the Mediterranean Sea and can provide assistance in studying the dynamics of POC in the Mediterranean. The curve corresponding to the TRRF product is relatively smooth, with POC concentrations mostly concentrated near the average value, while the curve corresponding to the NASA product is more erratic, with larger variations in POC concentration. This may be because the band ratio algorithm is more sensitive to sensor noise and atmospheric uncertainties and less capable of fitting nonlinear relationships than machine learning algorithms. It is more sensitive to changes in influencing factors, resulting in greater fluctuations in the estimated POC concentration.

**Figure 7:**
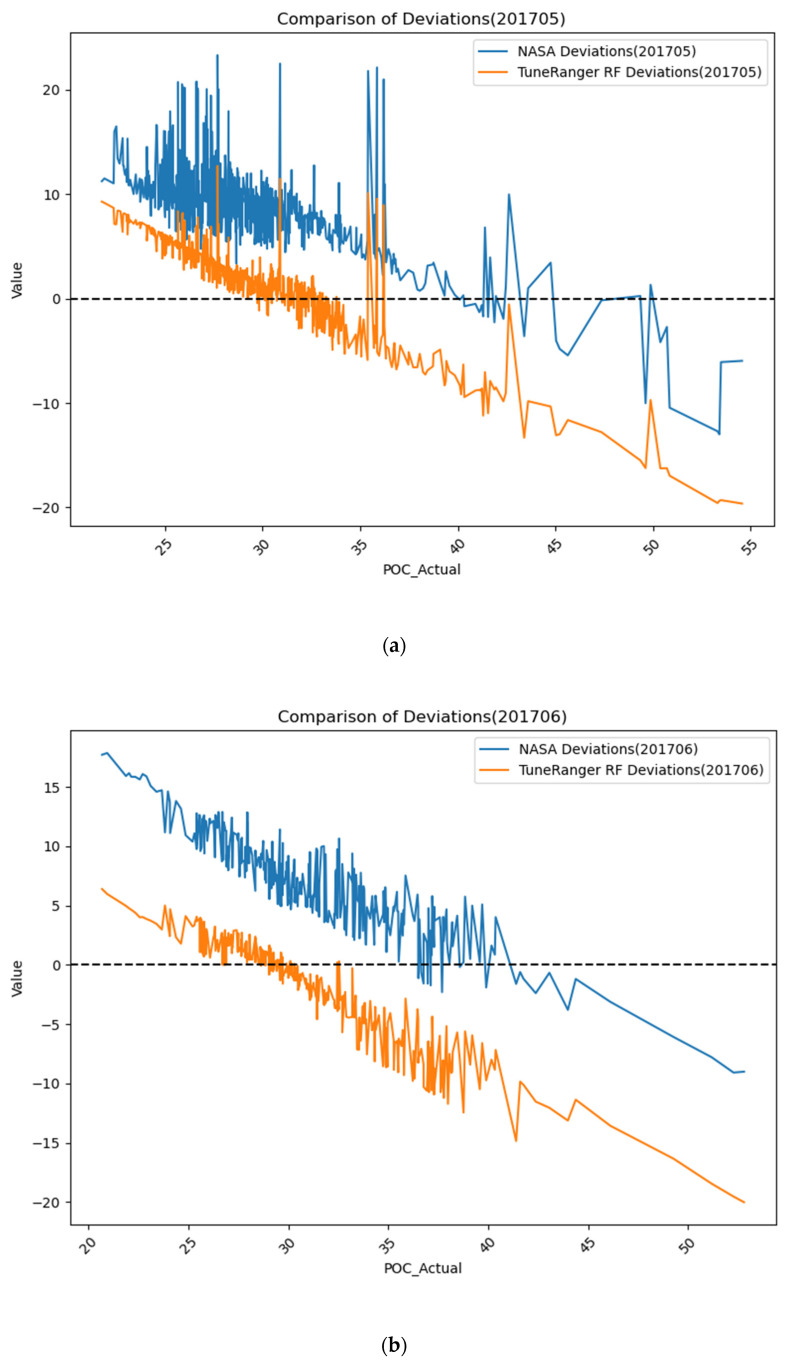

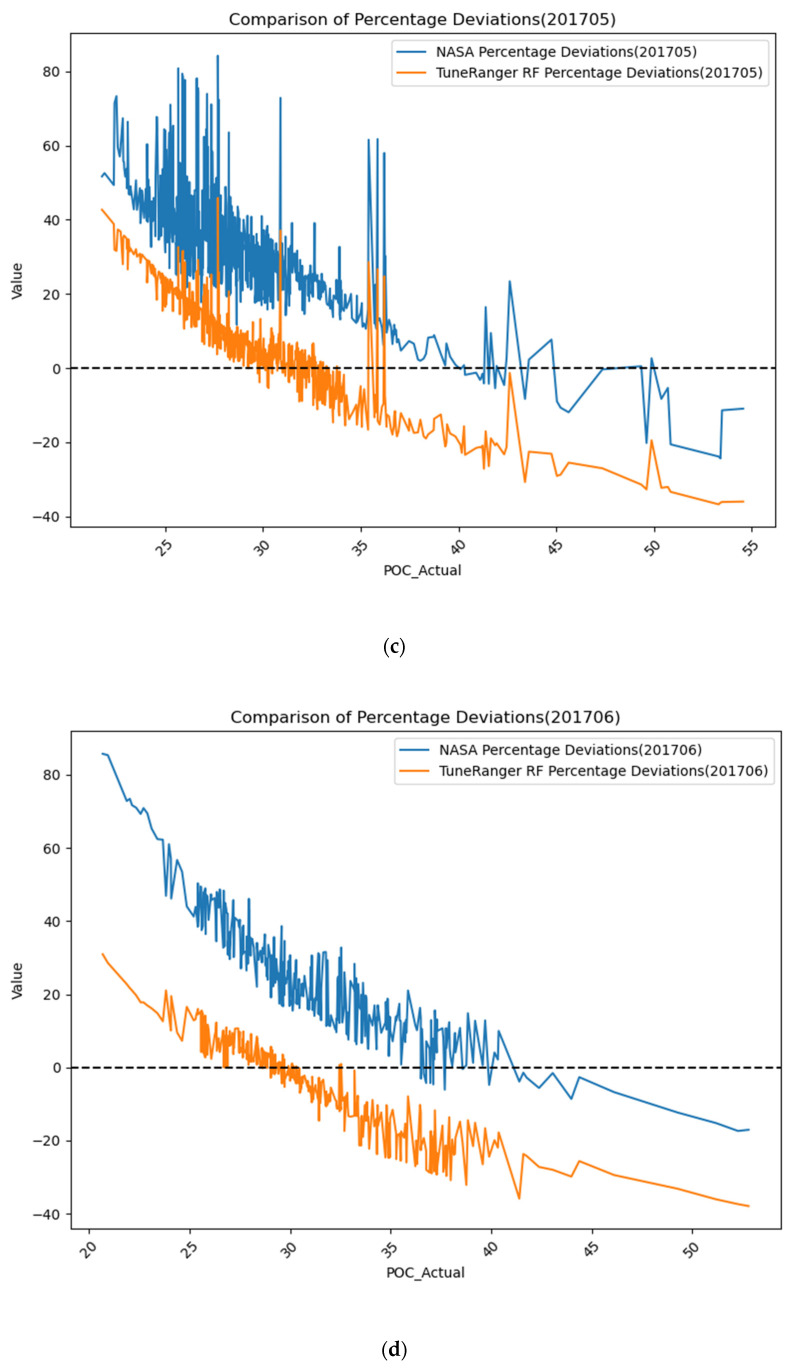
Deviations and percentage deviations between values obtained with random forest product optimized with tuneRanger R package and NASA product and actual measured values in May and June 2017 (Subfigures (**a**,**b**) show the result deviations between the NASA products and the TRRF products obtained in this study for May 2017 and June 2017, respectively. Subfigures (**c**,**d**) illustrate the percentage deviations between the NASA products and the TRRF products obtained in this study for May 2017 and June 2017, respectively).

To further compare the differences between the products obtained by the two algorithms and the measured values, the deviations and percentage deviations between the TRRF products, NASA products, and the measured values for May and June 2017 are shown in Figure 7. Figure 7 is a line plot with the measured POC values on the x-axis. It is evident in the graph that, at lower POC levels, both algorithms tended to overestimate the POC values to some extent, while at higher POC levels, they tended to underestimate the POC values. At lower POC values, the deviation of the TRRF from the measured values was smaller than that of NASA’s band ratio algorithm, which had not previously been observed. However, after POC exceeded approximately 40 mg/m^3^, the deviation of the TRRF algorithm from the measured values became larger than that of NASA’s band ratio algorithm. The reason may be that the POC data collected in this study were mostly concentrated in the range of 20–40 mg/m^3^, leading to an advantage in estimating POC within this concentration range. In the Mediterranean Sea, POC concentrations higher than 40 mg/m^3^ are mostly concentrated in coastal areas, where data collection is relatively limited.

#### 3.3.3. The Impact of Human Activities on the Distribution of Surface POC in the Mediterranean Sea

As depicted in Figure 4, the surface POC concentration in the Mediterranean Sea exhibited the characteristics of being higher in the west and north, lower in the east and south, higher near the coast, and lower far from the coast. It was found that nutrient levels influence microbial activities, thereby affecting the POC concentration. A stable isotope analysis revealed that 20% of the POM in the North and South China Seas comes from terrestrial inputs [41]. POC is a component of POM and is therefore also influenced by terrestrial inputs. Research has indicated that human activities in the Yangtze River Basin can significantly alter the coastal carbon cycle, thereby affecting the concentration of POC [42]. Therefore, regarding the distribution of the POC concentration in the Mediterranean Sea, the higher concentration near the coast and lower concentration far from the coast can be attributed to the significant influence of human activities in coastal areas, leading to increased terrestrial inputs of POC. The characteristics of higher concentrations in the west and north and lower concentrations in the east and south were analyzed based on the population density, GDP, and land use types of the coastal cities in the Mediterranean.

**Figure 8:**
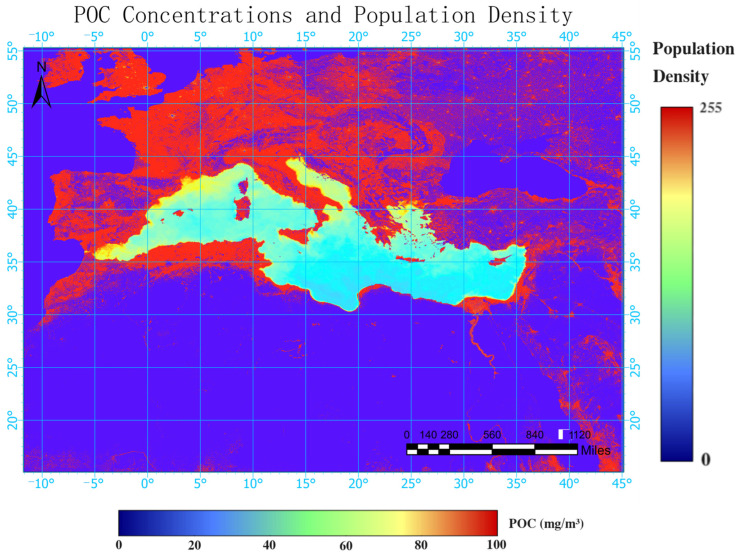
The particulate organic carbon concentration in the Mediterranean Sea and the coastal population density.

**Figure 9:**
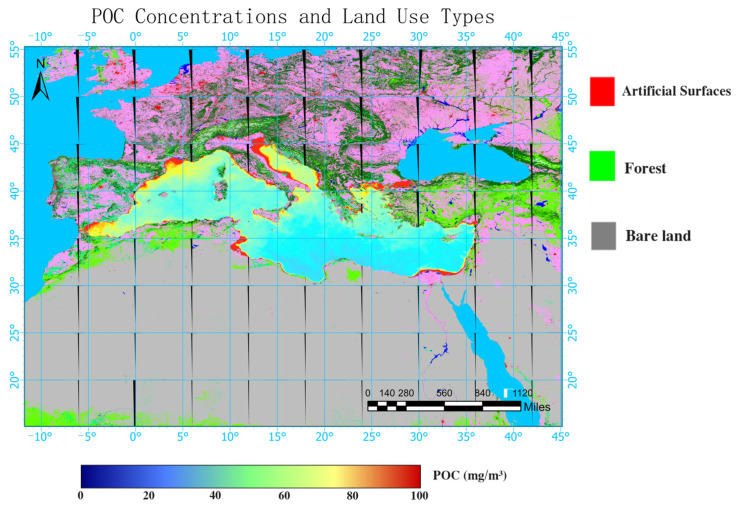
The particulate organic carbon concentrations and coastal land use types in the Mediterranean.

In Figure 8 and Figure 9, it can be observed that, in the north–south direction, the northern regions of the Mediterranean have higher population densities, including countries such as Spain, France, Italy, and Greece. The land use types in these regions are mostly artificially modified land, with some forested areas where vegetation covers more than 30% of the land. These countries also have relatively high GDPs. In contrast, the southern regions have lower population densities, including countries such as Egypt, Libya, Tunisia, Morocco, etc. The land use types in these regions are mostly barren lands or even deserts with vegetation covering less than 10% of the land. The GDPs of the cities in the south are lower than those in the north. Coastal areas are typically defined as regions within 40 miles of the coastline. For the Mediterranean, due to the specific natural resources and economic activities in the coastal areas, the population density along the Mediterranean coast is more than twice that of the entire Mediterranean region. In recent years, the tourism industry has rapidly developed along the northern coast of the Mediterranean. Currently, over 25% of the world’s hotels are located in the Mediterranean region. However, this development has also put significant environmental pressure on the Mediterranean [43]. While the GDP continues to rise, the commercial fishing and tourism industries are developing rapidly. The use of artisanal fishing and the exploitation of red coral have significantly impacted the biodiversity of the Mediterranean region. The increase in recreational activities also affects both species and habitats, and the arrival of nearly 120 million tourists each year further burdens the Mediterranean environment [44]. All these factors contribute to intensified eutrophication and influence microbial activities, resulting in increases in the surface POC concentrations in the coastal areas of the Mediterranean, which are higher near the coast and lower far from the coast. Population density, GDP, and land use types are robust indicators that reflect human activities. Figure 8 and Figure 9 show that cities in the western and northern regions of the Mediterranean have higher population densities, higher GDPs, and greater degrees of anthropogenic land development. This explains why the surface POC concentration in the Mediterranean exhibits the pattern of being higher in the west and north and lower in the east and south. Anna Maciejewska et al. studied the dynamics of marine carbon components and the factors affecting POC concentration variations [45]. They found that areas with higher POC concentrations frequently experienced dense phytoplankton blooms and that the concentration of POC in seawater is highly dependent on factors such as the pH value and chlorophyll concentration, which collectively influence the marine environment. Therefore, the spatial distribution characteristics of POC identified in this study also indicate that in areas near the Mediterranean coast and in the western Mediterranean, there is significant proliferation of phytoplankton and more severe water pollution. In contrast, areas farther from the coast and in the eastern Mediterranean have less phytoplankton growth and better water quality. Thus, by retrieving the POC in the Mediterranean through this study, it was possible to indirectly determine the survival conditions of phytoplankton in different regions of the Mediterranean, which reflect the water quality statuses of different marine areas. This will provide substantial assistance in maintaining a good marine environment in the Mediterranean.

## 4. Conclusions

In this study, the POC distribution on the surface of the Mediterranean Sea was investigated to understand the carbon cycle at the ocean basin scale. To estimate the surface POC in the Mediterranean, we selected relationships between apparent optical parameters, inherent optical parameters, water components, and POC concentrations. Algorithms were chosen through feature selection using GD, considering 47 factors belonging to three categories that are likely to affect the POC concentration. The ten factors most suitable for estimating the Mediterranean POC concentration were identified. The dataset was split into training, validation, and test sets using a 6:2:2 ratio. Models were trained using the BRF algorithm, Bayesian optimized XGBoost algorithm, AdaBoost algorithm, and BPNN algorithm. Among the four algorithms, the BRF algorithm performed the best, with a deviation of −0.001, a variance of 0.004, an R^2^ of 0.851, an RMSE of 0.025 log10 (mg/m^3^), and an MAPE of 1.268%, indicating high accuracy. To further enhance the BRF algorithm’s performance in estimating the surface POC in the Mediterranean, we performed parameter tuning using TR, which resulted in the TRRF model used in this research. Compared to the BRF, the TRRF was more accurate and faster. The evaluation metrics of the BRF model using Python were an R^2^ of 0.851, an MSE of 1.125 (mg/m^3^)^2^, and an MAE of 1.045 (mg/m^3^). After parameter tuning using TR, the evaluation metrics of the TRRF model improved significantly to an R^2^ of 0.868, an MSE of 1.119 (mg/m^3^)^2^, and an MAE of 1.040 (mg/m^3^). The resulting product was compared with the standard NASA products, and it was found that both the deviation and percentage deviation were small. Furthermore, a comparison was made between the TRRF products, NASA products, and actual measurements, considering factors such as the true values, averages, deviations, and percentage deviations. It was concluded that the TRRF products outperformed the NASA products in estimating the POC in the Mediterranean and that they can provide significant assistance when studying the dynamics of POC in the Mediterranean. Compared to the other models used in this study, the TRRF model performed significantly better and was the best method for predicting the POC in the Mediterranean. Therefore, we chose to use the TRRF model as the final model. Finally, using the TRRF, surface POC products for May 2017 and June 2017 were produced, revealing the spatial distribution characteristics of POC: higher in the north, west, and near the coast and lower in the south, east, and far from the coast. This study also discussed the impact of human activities on the surface POC concentration in the Mediterranean, indicating that intense human activities can significantly increase the POC concentration.

However, this study had some shortcomings that need improvement.

The observational data collected in this study were mostly concentrated in the range of 20–40 mg/m^3^ and lacked a sufficient number of samples with high POC concentrations. This resulted in the algorithm performing well at lower POC concentrations but performing poorly when estimating POC concentrations higher than 40 mg/m^3^. Therefore, in the future, more surface POC samples from the Mediterranean should be collected to increase the proportion of high-concentration POC samples and improve the accuracy of the model.

When exploring the impact of human activities on the spatial distribution of POC in the Mediterranean, this study qualitatively described the distribution based on maps. In the future, methods will be considered to quantitatively assess the influence of human activities on the POC distribution.

## Figures and Tables

**Table 1:** Statistical summary of the satellite and reanalysis data. (The data are sorted by spatial resolution in descending order. Those with the same spatial resolution are sorted in alphabetical order.).

Parameter	Full Name	Transducers/Product ID	Spatial Resolution	Time Resolution	Amount
BBP	backscattering coefficient of particles	OCEANCOLOUR_GLO_BGC_L3_MY_009_103	4 × 4 km	daily	37
CDM	volume absorption coefficient of radiative flux in seawater due to dissolved organic matter and non-algal particles	OCEANCOLOUR_GLO_B GC_L3_MY_009_103	4 × 4 km	daily	37
Kd_490	diffuse attenuation coefficient at 490 nm	OCEANCOLOUR_GLO_BGC_L3_MY_009_103	4 × 4 km	daily	37
Rrs (412, 443, 490, 555, and 670)	remote sensing reflectance (412 nm, 443 nm, 490 nm, 555 nm, and 670 nm)	OCEANCOLOUR_GLO_BGC_L3_MY_009_103	4 × 4 km	daily	37
Rrs (547, 645, 667, 469, 488, and 510)	remote sensing reflectance (547 nm, 645 nm, 490 nm, 488 nm, and 510 nm)	MODIS Aqua	4 × 4 km	daily	37
SPM	suspended particulate matter	OCEANCOLOUR_GLO_B GC_L3_MY_009_103	4 × 4 km	daily	37
SSTemp	sea-surface temperature	METOFFICE-GLO-SST-L4-REP-OBS-SST	0.05° × 0.05°	daily	37
SSS	seawater salinity	GLOBAL_MULTIY EAR_PHY_001_030	0.083° × 0.083°	daily	37
ZEU	euphotic zone depth	GLOBAL_MULTIYE AR_BGC_001_033	0.083° × 0.083°	daily	37
Dos	sea-surface density	MUTOBS_GLO_PHY_S_SURFACE_MYNRT_015_013	0.125° × 0.125°	daily	37
Chl	chlorophyll a	GLOBAL_MULTIY EAR_BGC_001_029	0.25° × 0.25°	daily	37
Mld	ocean mixed-layer thickness	MULTIOBS_GLO_PHY_TSUV_3D_MYNRT_015_012	0.25° × 0.25°	weekly	7
NO_3_	nitrate	GLOBAL_MULTIY EAR_BGC_001_029	0.25° × 0.25°	daily	37
O_2_	oxygen	GLOBAL_MULTIY EAR_BGC_001_029	0.25° × 0.25°	daily	37
pH	potential of hydrogen	cmems_mod_glo_bgc_my_0.25_P1M-m	0.25° × 0.25°	weekly	7
PO_4_	phosphate	GLOBAL_MULTIY EAR_BGC_001_029	0.25° × 0.25°	daily	37
SiO_3_	silicate	GLOBAL_MULTIY EAR_BGC_001_029	0.25° × 0.25°	daily	37
Ugos	geostrophic eastward ocean velocity	SEALEVEL_GLO_P HY_L4_MY_008_047	0.25° × 0.25°	daily	37
Vgos	geostrophic northward ocean velocity	SEALEVEL_GLO_P HY_L4_MY_008_047	0.25° × 0.25°	daily	37

**Table 2:** Accuracy of the models for the training, validation, and test sets (The bold text represents the metric values of the best-performing algorithm for each column of indicators in each dataset).

Dataset		Bias	Variance	R^2^	RMSE	MAPE
Training	BPNN	0.054	0.816	0.93	0.27	4.02%
	XGBoost	−3.040	0.005	**0.99**	**0.01**	**0.363%**
	BRF	−8.506	0.005	0.96	**0.01**	0.657%
	AdaBoost	**0.0036**	**0.0045**	0.74	0.034	1.941%
Validation	BPNN	−0.002	1.031	0.821	0.454	20.132%
	XGBoost	−0.0030	0.0049	**0.84**	0.028	**1.312%**
	BRF	**−0.0005**	**0.0033**	0.78	**0.027**	1.382%
	AdaBoost	−0.0037	0.0049	0.66	0.040	2.22%
Test	BPNN	**−0.0002**	0.005	0.821	0.030	1.547%
	XGBoost	0.004	**−0.0002**	0.831	**0.025**	1.288%
	BRF	−0.001	0.004	**0.851**	0.026	**1.284%**
	AdaBoost	0.002	0.004	0.631	0.037	2.088%

**Table 3:** Performance of different optimization methods for random forests.

RF Optimization Method	R^2^	MSE	MAE
BRF (Bayesian optimized random forest)	0.851	1.125	1.045
TRRF (tuneRanger random forest)	0.868	1.119	1.040

**Table 4:** Comparison of this study with the previous study.

Study	Features Selected	Model	R^2^
This Study	MNDCI(I), NDCI (443), Kd_490, BBP, NO_3_, O_2_, PO_4_, SPM, SSS, and Chl	TRRF	0.87
Previous Study	Rrs490/Rrs555, Rrs490/Rrs510, Rrs510/Rrs555, Kd (490), CI, and Chl	RF	0.73

## Data Availability

Data are contained within the article.
